# Overexpression of SMS in the tumor microenvironment is associated with immunosuppression in hepatocellular carcinoma

**DOI:** 10.3389/fimmu.2022.974241

**Published:** 2022-12-05

**Authors:** Lin Xiang, Longhuan Piao, Dong Wang, Li-Feng-Rong Qi

**Affiliations:** ^1^Department of Translational Medicine Research Institute, Jiangsu Yifengrong Biotechnology Co., Ltd., Nanjing, Jiangsu, China; ^2^Department of Orthopedic Surgery, Shengjing Hospital of China Medical University, Shenyang, Liaoning, China; ^3^Department of Histology and Embryology, Binzhou Medical University, Yantai, Shandong, China; ^4^State Key Laboratory of Natural Medicines, China Pharmaceutical University, Nanjing, Jiangsu, China

**Keywords:** hepatocellular carcinoma, spermine synthase, immune checkpoint blockade (ICB), polyamine metabolism, tumor immune microenvironment (TIME)

## Abstract

Disorders of polyamine metabolism may contribute to the development of hepatocellular carcinoma (HCC), but the precise mechanism remains unknown. This study reports that spermine synthase (SMS), an enzyme involved in polyamine biosynthesis, is overexpressed in HCC and not associated with hepatitis virus infection in HCC patients. The results of analyzing the clinical data of HCC patients showed that SMS level as a categorical dependent variable was related to clinicopathological features of poor prognosis. Furthermore, the Kaplan-Meier survival analysis and ROC curve indicated that increased SMS level is associated with poor survival rate in HCC and may be a potential biomarker to discriminate HCC tissues. However, SMS overexpression limited the therapeutic effect of immune checkpoint blockade (ICB), which seemed to be related to the immunosuppressive effect of the HCC immune microenvironment formed by higher mRNA transcript levels of immune checkpoints and higher infiltration levels of immunosuppressive cells. In samples with high and low SMS expression, functional enrichment analysis of the differentially expressed genes (DEGs) showed that SMS may be linked to the occurrence and development of HCC by affecting a variety of immune-related pathways, such as Intestinal immune network for IgA production, Fc gamma R-mediated phagocytosis, Antigen processing and presentation, Th1 and Th2 cell differentiation. Subsequently, analysis of the co-expression network of SMS in the liver hepatocellular carcinoma (LIHC) cohort revealed that SMS has a broad impact on multiple important immune- and metabolic-related processes in HCC. In summary, SMS is a promising biomarker to differentiate the prognosis, immune characteristics, and holds promise as a potential target for ICB therapy to improve HCC.

## Introduction

Liver cancer is a great health problem around the world and is estimated to affect more than 1 million individuals annually (1). Hepatocellular carcinoma (HCC), the most prevalent primary liver cancer, occurs easily in the context of chronic liver disease due to hepatitis virus infection, heavy alcohol intake or metabolic syndrome (2). Because of the limitations of early diagnosis technology, many HCC patients are confirmed as an advanced period at the initial diagnosis, losing the opportunity for healing by surgery or ablation (3). Currently, immune checkpoint blockade (ICB) has been admitted as an encouraging treatment modality for advanced HCC patients. Although ICB targeting the PD-1 and CTLA-4 have been approved for second-line therapy of HCC (4–6), response rates to ICB therapy only ranged from 15% to 30% (7–9). Because an immunosuppressive tumor microenvironment is promoted by the tumor cells, the infiltrating stromal and immune cells. Moreover, the capability of hepatoma cells to escape immune surveillance and potentially resist ICB treatment is likely due to the further enhancement of immunosuppression by tolerogenic liver environment (10). At present, the lack of clinically available biomarkers to assess response to ICB limits the efficacy and narrows the range of patients who could get help from ICB. Therefore, there is a pressing demand to develop effective biological targets to enhance the efficacy of ICB in HCC.

Liver has a central position in the metabolism of amino acids that contributes to biochemical processes indispensable for cell proliferation (11). There is a significant change of amino acids concentrations in specific bio-fluids from patients with liver diseases (12, 13). Recent studies have also shown the alterations of particular amino acids in diagnosed HCC patients (14–17).

In this study, we identified an amino acid metabolism-related hub gene, Spermine Synthase (SMS), an important regulator of polyamine metabolism, which was related to the prognosis of HCC patients from TCGA. Moreover, we investigated the effect of SMS expression on response to immunotherapy and analyzed its relationship with the degree of immune infiltration and gene transcription at immune checkpoints. Furthermore, we assessed SMS expression in the immune microenvironment in publicly available single-cell transcriptome sequencing data sets for HCC. Finally, we analyzed the differentially expressed genes (DEGs) in the different SMS expression samples and co-expression gene of SMS to explore the precise mechanisms of SMS in HCC. Our data implied that SMS is an encouraging biomarker to distinguish the prognosis, immune characteristics, and might act as a potential target for ICB therapy improvement in HCC.

## Materials and methods

### Identification of amino acid-metabolism related hub genes in TCGA-LIHC

In the GEPIA2 (18) (http://gepia2.cancer-pku.cn/) database, ANOVA and LIMMA methods were adopted to study the tumor and paired normal samples in the TCGA-LIHC dataset, respectively, to obtain a list of DEGs (with the |Log2FC| > 1 and q-value < 0.01). Intersection DEGs of the two lists above were screened out by Venn analysis. The comprehensive list of amino acid and derivative metabolic process was obtained from the Molecular Signatures Database v7.5.1 (MSigDB) (19–21) (https://www.gsea-msigdb.org/gsea/msigdb/index.jsp). Genes were annotated by the GO term GO:0006519. Intersection AAMRHGs of two gene lists above were screened out by Venn analysis. All hub genes made out by multivariate Cox regression analysis were conducted in TIMER2.0 (22–24) (http://timer.cistrome.org/).

### RNA sequencing data collection and analysis

RNA-sequencing expression (level 3) profiles and relevant clinical data for LIHC were obtained from the TCGA dataset (https://portal.gdc.com). The current-release (V8) GTEx datasets were downloaded from the GTEx data portal website (https://www.gtexportal.org/home/datasets). HCCDB database (25) (http://lifeome.net/database/hccdb) was also used in SMS expression analysis in HCC. The mRNA and Protein level of AAMRHBs in HCC and normal tissues were tested at “TCGA analysis” and “CPTAC analysis (26)” module of UALCAN (27) (http://ualcan.path.uab.edu/analysis-prot.html), respectively. In addition, cell line mRNA expression matrix of tumors was from the Cancer Cell Line Encyclopedia (CCLE) dataset (28) (https://portals.broadinstitute.org/ccle), which was used to validate AAMRHGs expression in cancer cell lines.

### Evaluation of the AAMRHGs prognostic signature

The HCC patients in this study were separated to two groups with high and low gene expression. The basis for the demarcation is the median value of gene. The clinical data analized by single-gene binary logistic regression was performed with AAMRHGs as the independent variable, low expression as the reference, and clinical characteristics as the dependent variable. The prognostic data for HCC patients were downloaded from the TCGA dataset.

The Kaplan-Meier (KM) survival curve analysis of overall survival (OS), disease-specific survival (DSS) and progression free survival (PFS) were executed by R package Survival and Survminer. timeROC (29) was adopted to analyze time-dependent receiver operating characteristic (ROC).

Univariate and multivariate cox regression analysis (29) were applied to determine the proper terms to establish the nomogram. Statistics of each variable were processed by “forestplot” R package to form forest map.

A nomogram was developed based on the results of multivariate cox proportional hazards analysis to predict the X-year overall recurrence. The nomogram provided a graphical representation of the factors which can be used to calculate the risk of recurrence for an individual patient by the points associated with each risk factor through ‘rms’ R package (30).

ROC curve analysis was executed by GraphPad Prism Version 9 to assess the discriminatory accuracy of SMS expression in predicting evaluated the extent to which they could separate each tumor entity from the normal tissue.

### Prediction of the therapeutic effect of ICB and analysis of immune function

We used TIDE (http://tide.Dfci.harvard.edu/) to evaluate the potential clinical efficacy of immunotherapy in different SMS overexpression groups (31, 32). We selected SIGLEC15, TIGIT, CD274, HAVCR2, PDCD1, CTLA4, LAG3 and PDCD1LG2 (33–36) as immune-checkpoint-relevant transcripts and extracted expression values of these genes from two groups. Immune infiltration level results between two groups were evaluated by immunedeconv (37) which integrates 6 latest algorithms, including TIMER (24), CIBERSORT (38), quanTIseq (39), xCell (40), MCP-counter (41) and EPIC (42). The results were obtained by TIMER2.0 (5). Results (p<0.001) were visualized by the Pheatmap package. Moreover, we used “gsva” package of ssGSEA to perform quantitative analysis of the immune cells and pathways between the high- and low-expression groups. 28 immune cell subpopulations were collected from the literature (43).

### Immune microenvironment analysis based on scRNA-seq expression

Single-cell RNA sequencing (scRNA-seq) was adopted to reveal the distribution and expression of AAMRHGs in the immune microenvironment of HCC. The data (GSE98638, GSE125449, GSE140228) was obtained from scTIME Portal (44) (http://sctime.sklehabc.com/), a database and a portal for single cell transcriptomes of tumor immune microenvironment. The portal implemented tumor immune microenvironment specific analysis modules to allow the data exploration. GSE98638 contains data obtained by deep single-cell RNA sequencing on single T cells isolated from peripheral blood, tumor and adjacent normal tissues from hepatocellular carcinoma patients. GSE125449 contains data obtained by single-cell transcriptome profiling of liver cancer biospecimens from nine hepatocellular carcinoma and ten intrahepatic cholangiocarcinoma patients. GSE140228 contains data obtained by single-cell transcriptome profiling of CD45^+^ immune cells for HCC patients from five immune-relevant sites (tumor, adjacent liver, hepatic lymph node (LN), blood, and ascites).

### Functional enrichment analysis

The limma package in the R software was adopted to study differentially expressed mRNA, threshold for which was set with reference to adjusted p < 0.05 and |Log2FC| > 1, between high- and low- expression groups. “ClusterProfiler (45)” package was adopted to carry out Gene ontology (GO) enrichment and Kyoto Encyclopedia of Genes and Genomes (KEGG) pathway analyses of co-expression genes. The GSEA was carried out using WebGestalt (WEB-based Gene Set Analysis Toolkit is a functional enrichment analysis web tool) (46). Then GSVA (47) was performed on Hallmark annotation libraries in MSigDB respectively to obtain the matrix of samples and pathways. Limma package was used for difference analysis between the high- and low- expression group. 19 important signaling pathways were collected from the literature (48), and the ssGSEA algorithm was adopted to predict the score of the corresponding pathway of each sample through the expression matrix, and then grouped according to the level of gene expression.

### Co-expression networks analysis

To identify gene co-expressed with the AAMRHGs, the LinkedOmics tool (49) (http://www.linkedomics.org) was adopted to calculate the Pearson’s correlation coefficient between AAMRHGs and others. Top 20 positively and negatively correlated genes were adopted to analyze their effect on OS of HCC. Top 50 positively and negatively correlated genes were used for GO and KEGG enrichment analyses.

### Statistical analysis

Statistical analyses were carried out using R version 4.0.3 and GraphPad Prism 9.0. Statistical differences of two groups and three groups were compared through the Wilcox test and Kruskal-Wallis test, respectively. Wilcoxon rank-sum test was adopted to analyze SMS expression in non-paired samples. Wilcoxon rank signed test was adopted to analyze SMS expression in paired samples. The log-rank test was adopted to analyze the differences between survival curves. p < 0.05 was considered statistically significant.

## Results

### Identification of amino acid and derivative metabolic process related genes from TCGA-LIHC and GEO databases

**Figure 1:**
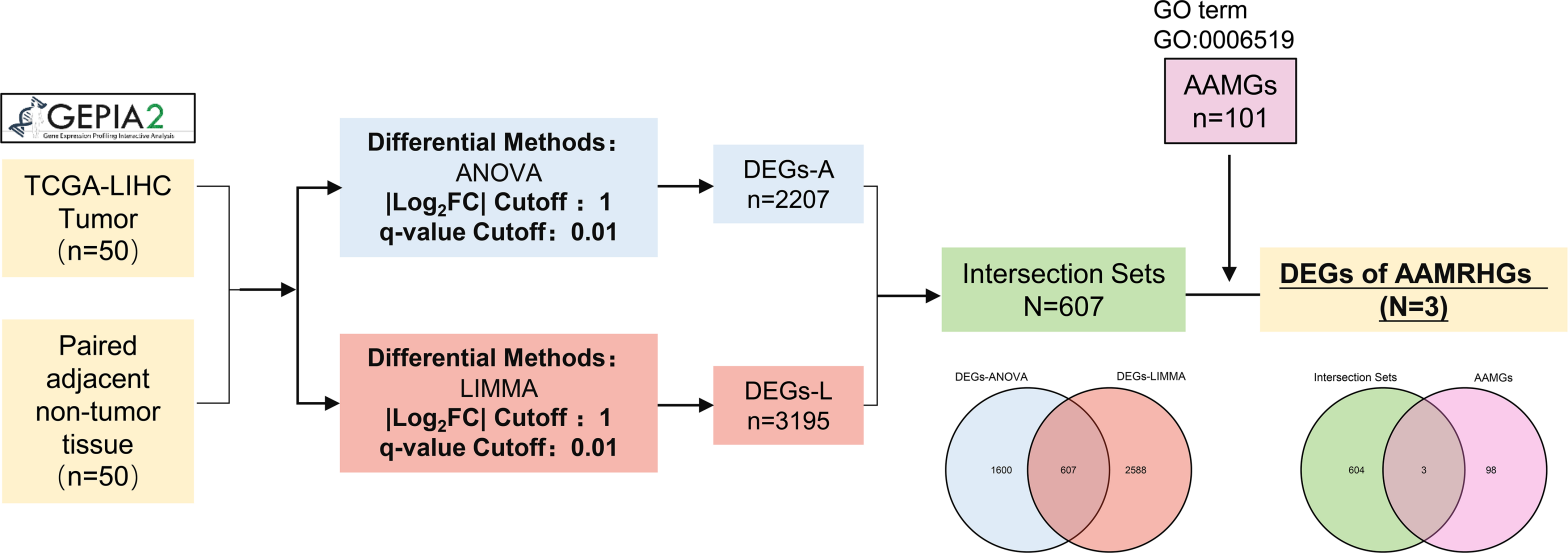
Flow chart of identifying target genes for study. GEPIA2 picture is from http://gepia2.cancer-pku.cn/.

This research was carried out in accordance with the flow chart exhibited by **Figure 1**. 2207 and 3195 DEGs between the tumor and normal groups were identified in the TCGA-LIHC gene expression profiles obtained by ANOVA and LIMMA algorithms, respectively. Furthermore, 607 genes that overlap in two algorithm groups were shown by a venn diagram. And then, intersection of 607 DEGs and AAMGs (GO:0006519) yielded 3 amino acid and derivative metabolic process related differentially expressed genes (AAMRHGs), included in the subsequent functional classification analysis (**Figure 1**). Single gene or a gene group suitable for research was determined by multivariate Cox regression analysis in Tumor Immune Estimation Resource 2.0 (TIMER2.0) Database. Finally, SMS was selected (**Extended Data Figures S1A, B**).

### Upregulation of SMS in HCC

**Figure 2:**
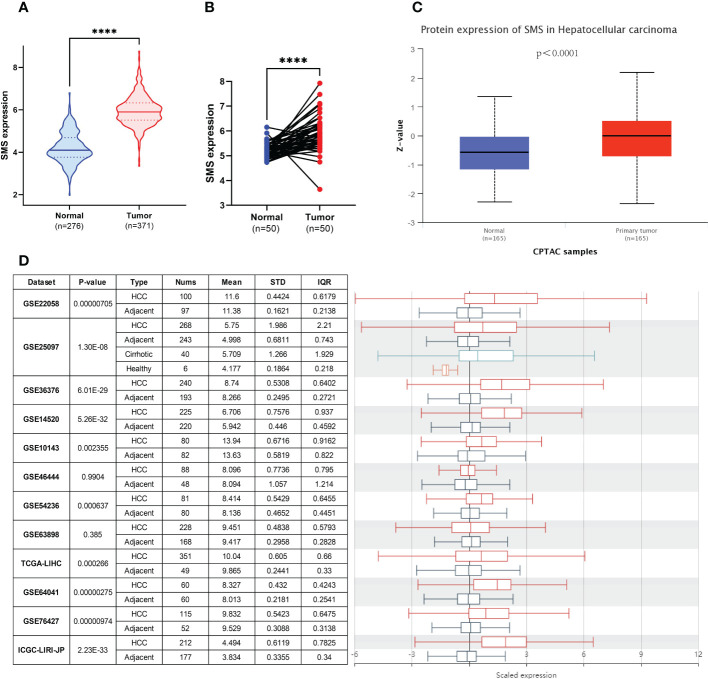
Upregulation of SMS in HCC. **(A)** SMS mRNA expression in 371 HCC samples (371 in TCGA-LIHC) and 276 normal samples (50 in TCGA-LIHC dataset and 226 in GTEx dataset). **(B)** SMS mRNA expression in 50 HCC and paired normal samples. **(C)** SMS protein expression based on CPTAC. **(D)** Comparison of the transcriptional level of SMS between HCC tissues and paired normal tissues in HCCDB. ****p < 0.0001 vs. indicated control.

We compared SMS expression in 371 HCC tumor tissues and 276 normal tissues (50 in TCGA-LIHC dataset and 226 in GTEx dataset) by Wilcoxon rank sum test. The expression levels of SMS were markedly higher in tumor tissues compared to unpaired (p < 0.001; **Figure 2A**) and paired (p < 0.001; **Figure 2B**) normal tissues, respectively. To clarify the transcriptional and translational levels of SMS in HCC tissues, we carried out CPTAC analysis of UALCAN and analysis of 11 HCC study cohorts in the HCCDB database, respectively. We found that both the protein (**Figure 2C**) and mRNA (**Figure 2D**) expressions of SMS were pronouncedly elevated in HCC tissues relative to normal tissues. There were also consistent trends in the mRNA and protein levels of SMS in pan-cancer tissues by analysis of the pan-cancer study cohorts of UALCAN (**Extended Data Figures S2A, B**). As revealed by Cancer Cell Line Encyclopedia (CCLE) analysis, SMS expression in Liver cancer cell lines were higher than the average level of pan-cancer cell line (**Extended Data Figures S2C, D**). These results illustrate a strong association between SMS upregulation and HCC tumorigenesis.

### Evaluation of the prognostic relevance of SMS in HCC

To make clear the role and prominence of SMS expression, the clinicopathological features of HCC patients with different SMS expression were investigated (**Table 1**). Contrast to the low expression of SMS group, patients in the high expression of SMS group had a significantly higher ratio of more severe primary tumor (T) stage, more severe lymph nodes (N) stage, more severe metastasis (M) stage, worse Pathologic stage, Tumor status, and higher Alpha-fetoprotein (AFP), all which with significant difference.

**Table 1:** Clinical characteristics of HCC patients in TCGA database.

Characteristic	Low expression of SMS	High expression of SMS	P value
n	185	186	
T stage, n (%)			0.010
T1	106 (28.8%)	75 (20.4%)	
T2	40 (10.9%)	54 (14.7%)	
T3	32 (8.7%)	48 (13%)	
T4	5 (1.4%)	8 (2.2%)	
N stage, n (%)			0.623
N0	123 (48%)	129 (50.4%)	
N1	1 (0.4%)	3 (1.2%)	
M stage, n (%)			0.627
M0	123 (45.6%)	143 (53%)	
M1	1 (0.4%)	3 (1.1%)	
Pathologic stage, n (%)			0.013
Stage I	100 (28.8%)	71 (20.5%)	
Stage II	39 (11.2%)	47 (13.5%)	
Stage III	33 (9.5%)	52 (15%)	
Stage IV	2 (0.6%)	3 (0.9%)	
Tumor status, n (%)			0.132
Tumor free	108 (30.7%)	93 (26.4%)	
With tumor	68 (19.3%)	83 (23.6%)	
Gender, n (%)			0.853
Female	59 (15.9%)	62 (16.7%)	
Male	126 (34%)	124 (33.4%)	
Age, median (IQR)	61 (53, 69)	61 (51, 69)	0.549
Height, median (IQR)	168 (161, 175)	167 (161, 173)	0.141
BMI, median (IQR)	24.84 (21.78, 29.34)	24.19 (21.7, 27.88)	0.257
Weight, median (IQR)	72 (61, 86.5)	68 (59, 78)	0.037
AFP(ng/ml), median (IQR)	7 (3, 48)	30 (8, 1836)	< 0.001
Albumin(g/dl), median (IQR)	4 (3.5, 4.38)	4 (3.5, 4.3)	0.629
Prothrombin time, median (IQR)	1.1 (1, 9.78)	1.1 (1, 3.62)	0.106

The univariate analysis with Logistic regression demonstrated that SMS expression was related to poor prognostic clinicopathological features (**Table 2**). Elevated SMS expression in HCC is positively associated with T stage, Pathologic stage, Histologic grade, Adjacent hepatic tissue inflammation, AFP and Fibrosis ishak score, meanwhile negatively associated with Race, Weight and Height significantly (all p < 0.05).

**Table 2:** Single-gene binary logistic regression.

Characteristics	Total (N)	Odds Ratio (OR)	P value
T stage (T2&T3&T4 vs. T1)	368	1.846 (1.223-2.799)	0.004
N stage (N1 vs. N0)	256	3.097 (0.391-63.065)	0.330
M stage (M1 vs. M0)	270	66367660.351 (0.000-NA)	0.994
Pathologic stage (Stage II&Stage III&Stage IV vs. Stage I)	347	1.768 (1.157-2.712)	0.009
Tumor status (With tumor vs. Tumor free)	352	1.362 (0.892-2.084)	0.153
Gender (Male vs. Female)	371	1.033 (0.669-1.596)	0.883
Race (Black or African American&White vs. Asian)	359	0.593 (0.389-0.902)	0.015
Age (>60 vs. <=60)	370	1.067 (0.710-1.606)	0.755
Weight (>70 vs. <=70)	344	0.626 (0.408-0.957)	0.031
Height (>=170 vs. < 170)	339	0.638 (0.412-0.984)	0.043
BMI (>25 vs. <=25)	335	0.778 (0.505-1.195)	0.252
Residual tumor (R1&R2 vs. R0)	342	2.208 (0.836-6.473)	0.122
Histologic grade (G3&G4 vs. G1&G2)	366	2.257 (1.464-3.507)	<0.001
Adjacent hepatic tissue inflammation (Mild&Severe vs. None)	234	1.829 (1.083-3.111)	0.025
AFP(ng/ml) (>400 vs. <=400)	278	3.309 (1.841-6.142)	<0.001
Albumin(g/dl) (>=3.5 vs. <3.5)	297	1.341 (0.780-2.333)	0.292
Prothrombin time (>4 vs. <=4)	294	0.529 (0.314-0.879)	0.015
Child-Pugh grade (B&C vs. A)	239	0.905 (0.368-2.186)	0.825
Fibrosis ishak score (1/2&3/4&5/6 vs. 0)	212	2.154 (1.199-3.953)	0.011
Vascular invasion (Yes vs. No)	315	1.306 (0.821-2.083)	0.261

**Figure 3:**
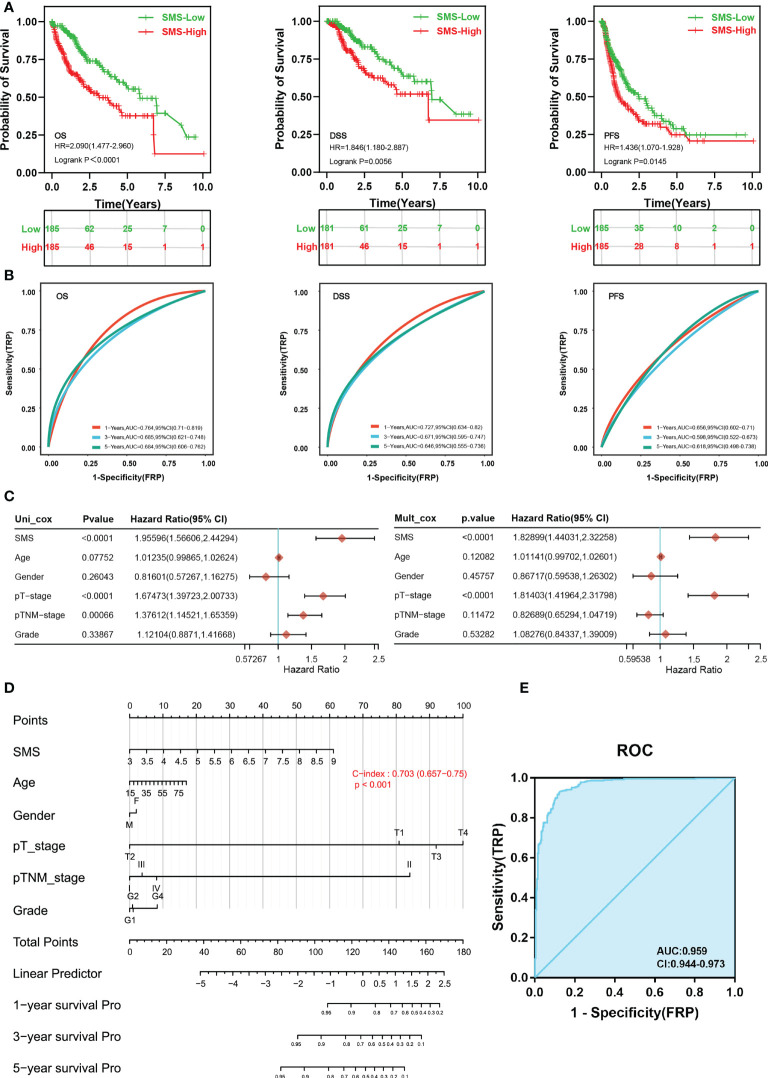
Differential SMS expression as a prospective biomarker of poor prognosis and to discriminate HCC samples. **(A)** OS, DSS and PFS of SMS mRNA in HCC cohort. **(B)** Time-dependent ROC analysis of SMS expression in HCC. **(C)** Forrest plot of univariate and multivariate Cox regression analysis in HCC. **(D)** Nomogram of SMS and other prognostic factors in HCC. **(E)** ROC curve assessing the performance of SMS for HCC diagnosis.

Next, Kaplan-Meier survival analysis illustrated that patients in the high expression of SMS group had remarkably shorter overall survival [OS, n=370, HR=2.090(1.477-2.960), log-rank P<0.001], disease-specific survival [DSS, n=362, HR=1.846(1.180-2.887), log-rank P=0.0056] and progression free survival [PFS, n=370, HR=1.436(1.070-1.928), log-rank P=0.0145] contrast to the low expression of SMS group in HCC (**Figure 3A**). To examine the ability of SMS to predict HCC development, through time-dependent ROC analysis, we found that SMS could predict the OS, DSS and PFS of HCC patients at 1, 3, and 5 years to a certain extent, whose AUC were mostly between 0.6-0.8 (**Figure 3B**). Univariate and multivariate Cox regression analysis were used to assess the independent prognostic value of 6 clinicopathological variables, including SMS, Age, Gender, pT stage, pTNM stage and grade, in terms of OS of HCC. SMS and pT stage were independent prognostic markers for OS (**Figure 3C**). Then we adopted a nomogram model for estimating these clinicopathological variables. The concordance index for this nomogram was 0.703 (0.657-0.75) (**Figure 3D**). Furthermore, SMS expression indicated discriminative effect with an AUC value of 0.956 (CI = 0.944-0.973) to identify tumors from normal tissue (**Figure 3E**). The result showed that SMS could be a heartening biomarker to discriminate HCC tissues. Those results imply that SMS expression foresees detrimental outcomes and is related to disease development in HCC patients.

### Correlation of SMS expression with ICB response and immune infiltration in HCC

**Figure 4:**
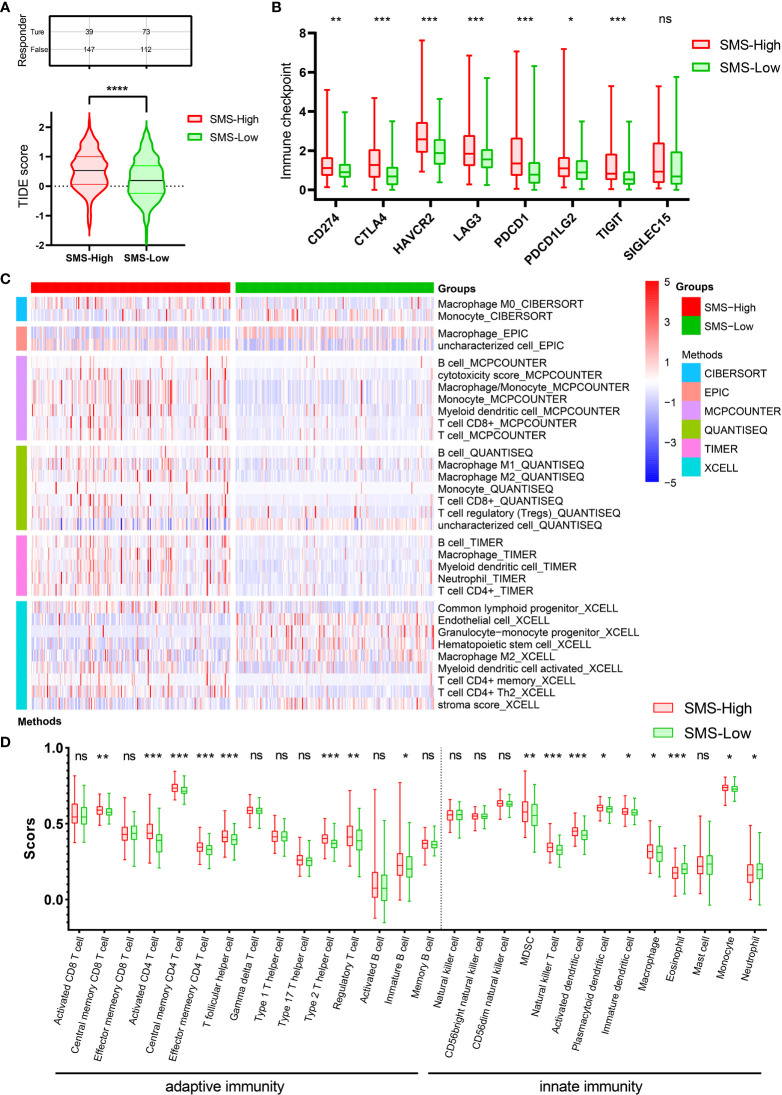
ICB immunotherapy response predict and Immune infiltration analysis. **(A)** Differences in TIDE prediction score between the indicated groups. **(B)** Expression of immune checkpoints between the indicated groups. **(C)** Heatmap of immune responses between the indicated groups. **(D)** ssGSEA immune cells scores between the indicated groups in boxplots. *p<0.05, **p<0.01, ***p<0.001, ****p<0.0001 vs. indicated control, ns, no significance.

Immunotherapy has revolutionized treatment of cancers. Although a subset of patients with HCC benefit from ICB therapy, many patients do not achieve significant benefit. We next examined whether SMS expression could predict ICB clinical response by Tumor Immune Dysfunction and Exclusion (TIDE) analysis. The result displayed a higher median TIDE score in SMS overexpression group, indicating a weakened ability to respond to ICB (**Figure 4A**). In addition, TIDE prediction score is also negatively correlated to anti-PD1 and anti-CTLA4 treatment effects. This prompted us to explore the relationship among SMS expression, immune checkpoint, and immune cell infiltration level. The mRNA transcription levels of immune checkpoints were generally increased in HCC with the higher SMS expression, suggesting that more intense immunosuppression was prevalent in HCC with higher SMS expression (**Figure 4B**).

The number and proportion of infiltrating immune cells play a principal role in cancer development and immunotherapy response, and thus correlate with patient prognosis. The association between SMS expression and immune infiltration in HCC was shown in the heatmap, which was analyzed by the R package immunodeconv (p<0.001, **Figure 4C**). Comparative analysis of immune cells affirmed that there were differences in various immune cells between two groups, such as Immature B cell, Immature dendritic cell, Macrophage, etc. (**Figure 4D**).

The results insinuate that strong immunosuppressive effect in the tumor microenvironment is a prerequisite for poor prognosis and tumor development in SMS-overexpressing HCC patients. In summary, SMS overexpression might affect immune cell infiltration in HCC patients, resulting in poor ICB response and efficacy against HCC.

### SMS expression in the tumor immune microenvironment of HCC

**Figure 5:**
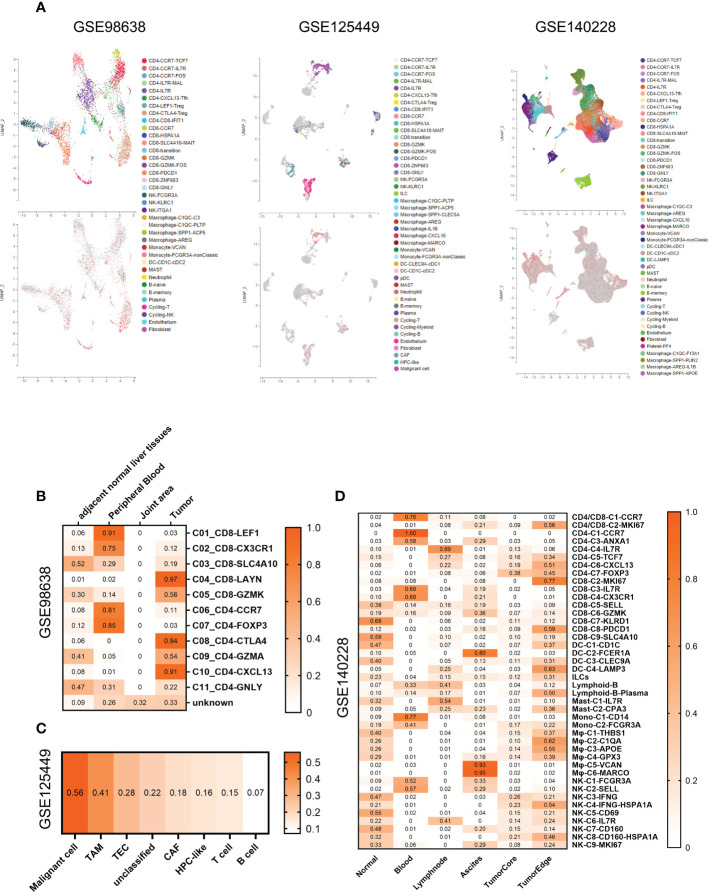
SMS expression within the tumor immune microenvironment of HCC. **(A)** UMAP annotated with cell types (Up panel) and colored by SMS positive cells (red) and none (gray) (Down panel). **(B–D)** Heatmaps of SMS gene expression positivity rates for each immune cell subset in GSE98638 **(B)**, GSE125449 **(C)**, and GSE140228 **(D)**.

To understand the impact of SMS gene expression on the tumor immune microenvironment, we acquired 3 HCC single-cell transcriptome datasets in the scTIME Portal (http://sctime.sklehabc.com/) and analyzed them in each dataset. Cells expressing SMS gene were red, while cells lacking SMS gene were gray (**Figure 5A**). In dataset GSE98638, compared with immune microenvironments derived from other tissues, T cell exhaustion and inflammatory senescence-related cells in tumor tissue had a higher positive rate of SMS expression (**Figure 5B**). In dataset GSE125449, malignant tumor cells and TAM tumor-associated macrophages were more positive than other cells in the immune microenvironment (**Figure 5C**). In dataset GSE140228, compared with immune microenvironments derived from other tissues, there were differences in SMS-expressing cells in the outer edge of tumor tissue, especially macrophages. In particularly, there were tumor-derived macrophages that express SMS in ascites fluid, which may be partly associated with cancer metastasis (**Figure 5D**). Overall, SMS expression is associated with a stronger tumor immunosuppressive effect in tumor immune microenvironment of HCC.

### Differential gene expression profiles comparing SMS-low and SMS-high in HCC

**Figure 6:**
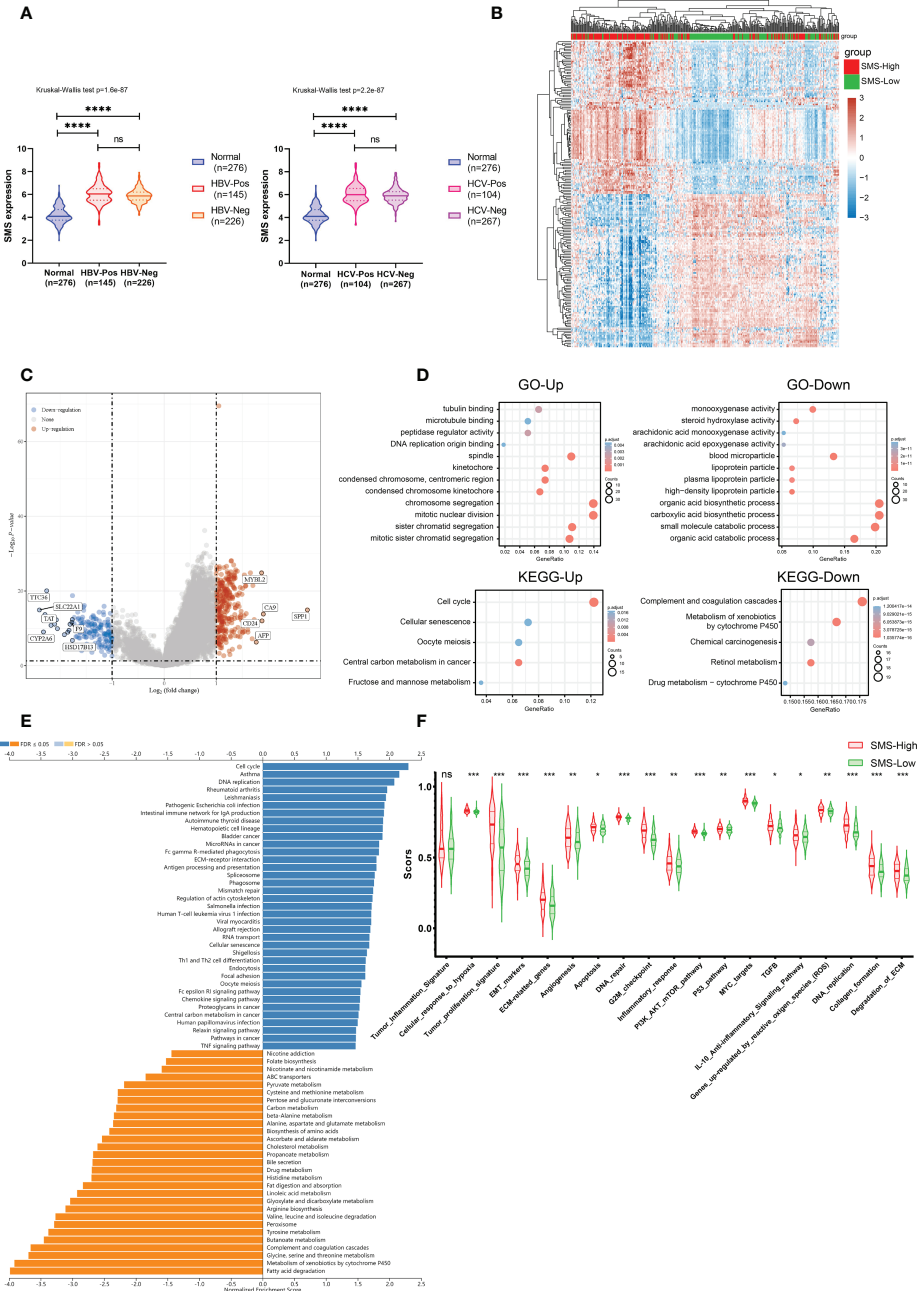
Functional analysis of DEGs in HCC patients with distinct SMS levels. **(A)** SMS expression in HCC patients with or without Hepatitis virus infection and in normal liver tissues. (Left, HBV; Right, HCV) **(B)** The heatmap of differential gene expression between the indicated groups. **(C)** Volcano plots of the gene expression profile data. **(D)** Up- and down-regulated genes were analyzed for GO terms and KEGG pathway. **(E)** GSEA of the high and low SMS expression clusters. **(F)** ssGSEA scores of 19 functional pathways related to cancer between the indicated groups. *p<0.05, **p<0.01, ***p<0.001, ****p < 0.0001 vs. indicated control, ns, no significance.

To exclude the effect of hepatitis infection on SMS expression, we examined the SMS expressions of tumors among the HCC patients with or without viral hepatitis in the TCGA-LIHC data set. There was no correlation between SMS expression and hepatitis B virus or hepatitis C virus infection (**Figure 6A**).

To find out the underlying mechanisms of SMS that facilitate tumor development, we analyzed DEGs in the high- and low-SMS expression samples. Microarray data was standardized by limma package. The clustering analysis and expression of DEGs were shown in a heatmap (**Figure 6B**) and a Volcano Plot (**Figure 6C**), respectively. According to the results of Gene ontology (GO) and Kyoto Encyclopedia of Genes and Genomes (KEGG) pathway analysis, we found that the DEGs were mostly enriched in chromosome segregation, mitotic nuclear division, organic and carboxylic acid biosynthetic process, etc. (**Figure 6D**).

Further, through GSEA by the WebGestalt, we assessed the downstream pathways for SMS. We found immune-related pathway enrichment results through KEGG analysis under the Functional Database option, such as Intestinal immune network for IgA production, Fc gamma R-mediated phagocytosis, Antigen processing and presentation, Th1 and Th2 cell differentiation. (**Figure 6E**). The results suggest that the abnormal expression of SMS may participate in the formation of immunosuppressive function in the immune microenvironment of HCC through these immune-related pathways.

In order to determine the relationship between SMS and cancer-related functional pathways, we calculated the functional pathway scores of 19 cancer-related functional pathways between the high and low expression groups of SMS by ssGSEA algorithm. As the results show, Tumor inflammation signature was similar between two groups. However, in SMS-high expression group, there were significant more active in tumor-related signaling pathways, such as Cellular response to hypoxia, Tumor proliferation signature, EMT markers, etc. (**Figure 6F**). These results suggest that SMS also may affect the occurrence and progression of HCC by regulating a variety of important tumor-related signaling pathways.

### Co-expression networks of SMS in HCC

**Figure 7:**
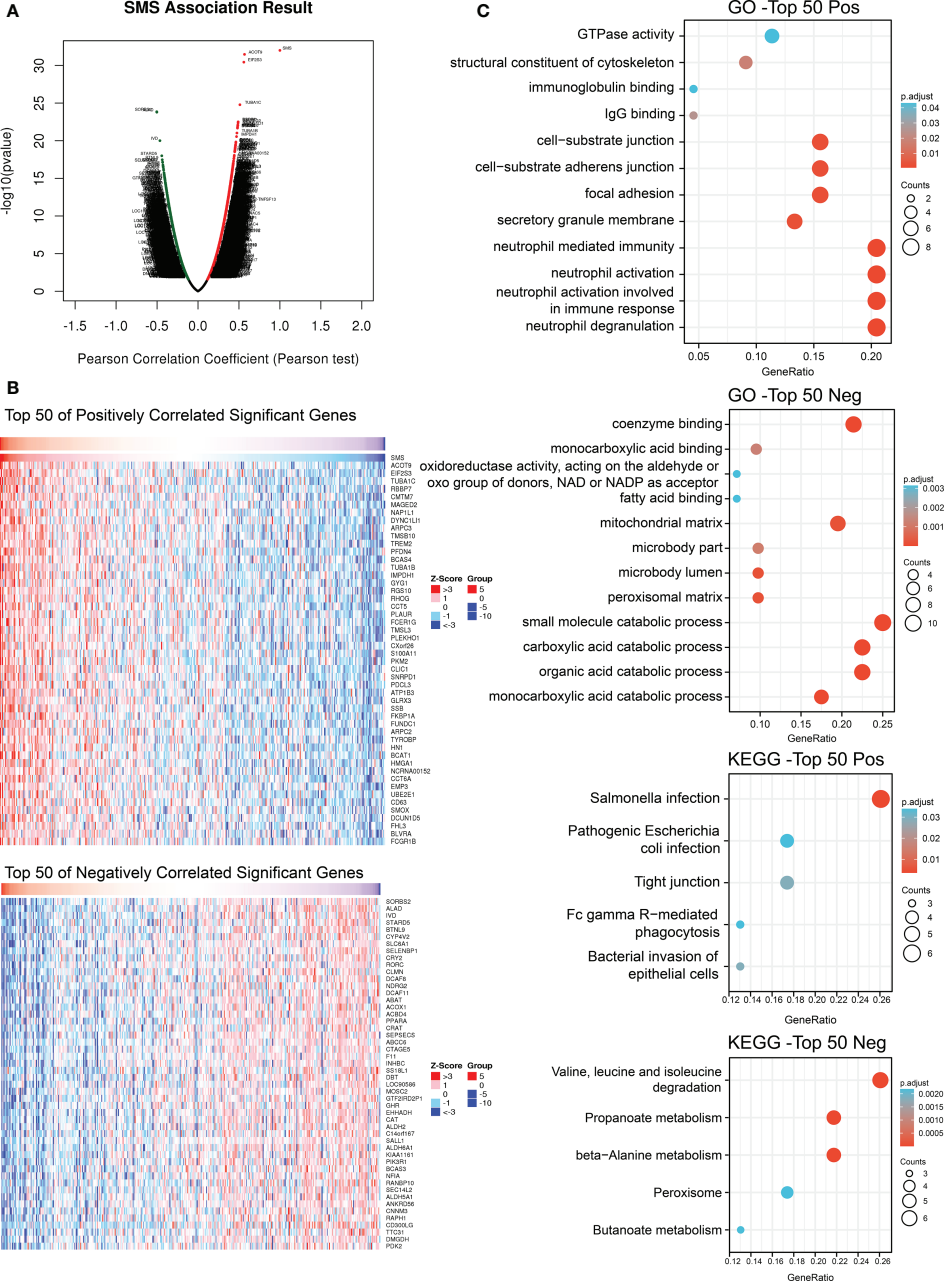
Genes co-expressed with SMS in HCC. **(A)** Co-expressed genes with SMS in HCC. **(B)** Indicated co-expressed genes co-transcript with SMS in HCC. **(C)** Indicated co-expressed genes with SMS were analyzed for GO terms and KEGG pathway.

Analyzing the co-expression network of SMS in the LIHC cohort by Linkedomics, we expected to reveal gene interactions and thereby mine SMS gene function. There were 12,073 genes and 7,842 genes that had significant positive and negative correlations with SMS, respectively (false discovery rate, FDR < 0.01) (**Figure 7A**). Then, from the above two correlation groups, the top 50 genes were selected to make a heat map display (**Figure 7B**). SMS expression was extremely positively correlated with ACOT9 (positive rank #1, r = 0.568, p = 3.38E-32), EIF2S3 (r = 0.560, p = 3.65E-31) and TUBA1C (r = 0.512, p = 1.66E-25).

Next, we assessed high- or low-risk genes by hazard ratios (HR) in the HCC overall survival analysis. The results showed that the top 20 positively correlated co-expressed genes and the top 20 negatively correlated co-expressed genes may be high-risk genes and low-risk genes of HCC, respectively (p < 0.05, **Table 3**). Therefore, SMS is likely to promote HCC progression by regulating the risk factors of HCC.

**Table 3:** Overall survival analysis of the top 20 genes positively and negatively correlated with SMS in HCC.

Neg Genes	HR	Log-rank P	Pos Genes	HR	Log-rank P
SORBS2	0.50 (0.35-0.71)	0.000	ACOT9	1.29 (0.91−1.82)	0.150
ALAD	0.72 (0.51-1.02)	0.064	EIF2S3	1.67 (1.18−2.36)	0.004
IVD	0.59 (0.42−0.84)	0.003	TUBA1C	1.99 (1.40−2.81)	0.000
STARD5	0.57 (0.40−0.80)	0.001	RBBP7	1.56 (1.10−2.20)	0.011
BTNL9	0.53 (0.38−0.76)	0.000	CMTM7	1.80 (1.27−2.54)	0.001
CYP4V2	0.61 (0.43−0.86)	0.004	MAGED2	1.44 (1.02−2.04)	0.036
SLC6A1	0.67 (0.47−0.94)	0.020	NAP1L1	1.70 (1.21−2.41)	0.002
SELENBP1	0.78 (0.55−1.11)	0.161	DYNC1LI1	2.15 (1.52−3.04)	0.000
CRY2	0.69 (0.49−0.97)	0.032	ARPC3	1.50 (1.06−2.12)	0.021
RORC	0.64 (0.45−0.90)	0.011	TMSB10	1.65 (1.17−2.33)	0.005
CLMN	0.85 (0.60−1.20)	0.352	TREM2	1.44 (1.02−2.03)	0.038
DCAF8	1.00 (0.71−1.41)	0.998	PFDN4	1.78 (1.26−2.51)	0.001
NDRG2	0.69 (0.49−0.98)	0.033	BCAS4	1.62 (1.15−2.29)	0.006
DCAF11	0.85 (0.60−1.20)	0.361	TUBA1B	1.56 (1.10−2.21)	0.011
ABAT	0.56 (0.40−0.79)	0.001	IMPDH1	2.15 (1.52−3.04)	0.000
ACOX1	0.79 (0.56−1.12)	0.180	GYG1	1.80 (1.27−2.54)	0.001
ACBD4	0.82 (0.58−1.16)	0.254	RGS10	1.56 (1.10−2.19)	0.013
PPARA	1.02 (0.72−1.44)	0.919	RHOG	1.54 (1.09−2.17)	0.016
CRAT	0.88 (0.62−1.24)	0.469	CCT5	1.61 (1.13−2.27)	0.007
SEPSECS	0.71 (0.50−1.00)	0.049	PLAUR	1.55 (1.10−2.18)	0.013

Finally, the top 50 positively correlated genes and the top 50 negative correlated genes were used for GO and KEGG enrichment analysis respectively. The result indicated that SMS co-expressed genes were chiefly related to neutrophil immune response pathway and organic matter catabolism process (**Figure 7C**). KEGG pathway analysis indicated that co-expressed genes were chiefly enriched in the Salmonella infection and Valine,leucine and isoleucine degradation (**Figure 7C**). These results suggest that SMS may be involved in immune-related processes in HCC.

## Discussion

Liver is the principal organ of amino acid metabolism. Alterations in amino acid metabolism are characteristics of HCC (50). In the present study, we screened an amino acid metabolism-related SMS gene in TCGA-LIHC dataset. SMS catalyzes the final step in the production of spermine from arginine and plays a key role in maintaining normal spermine levels (51, 52). Disordered expression of SMS has been found in colorectal cancer and breast cancer (53, 54). Analysis of multiple cancer clinical research datasets showed that aberrant SMS expression is a common phenomenon in most cancers. In this research we focused on liver cancer. Based on in-depth mining of RNA-seq data combined with relevant clinical information of HCC, we observed that the elevated SMS expression was related to poor prognosis of HCC patients. Moreover, this effect of SMS was independent of other factors through Single-gene binary logistic regression analysis and COX regression analysis. According to the timeROC curve analysis, we believed that SMS expression could help to infer the survival rate of HCC patients. Meanwhile, SMS expression raised with HCC tumor stage development, implying that SMS is related to HCC development. Furthermore, when assessing the sensitivity and specificity of SMS expression in predicting HCC tissue from normal tissue by ROC curve, it was found that the predictive capability of variable SMS was accurate in predicting tumor and normal prognosis (AUC = 0.959, CI = 0.944-0.973). Taken together, we speculate that the high expression of SMS in HCC patients would cause spermine accumulation, which is confirmed in the urine and plasma of cancer patients (55). Therefore, SMS might be employed as prospective biomarkers for diagnosis and prognostication of clinical consequences for HCC.

Owing to the limitations of HCC diagnostic techniques, patients often lose the opportunity for thoroughgoing treatment such as surgery or liver transplantation (3). Therefore, ICB therapy gives hope of cure for patients with advanced HCC (10). To evaluate the effect of SMS expression on the efficacy of ICB, we used the TIDE algorithm and discovered that TIDE score increased in the high-expressing SMS group. TIDE score is positively correlated with the incidence of immune escape, which may account for the poor response to ICB therapy (32). Through public data mining, we found that mRNA transcription level of immune checkpoints was generally increased in HCC with the higher SMS expression. Moreover, SMS expression was shown to be associated with HCC immune infiltration. Evidence suggests that SMS can be a potential biomarker for predicting response to ICB therapy.

Various stromal cells, cytokines, chemokines, etc. in the tumor microenvironment can regulate tumor development and have become potential therapeutic targets. Using publicly available hepatocellular carcinoma single-cell transcriptomic data analysis, we assessed the distribution of SMS expression in the immune microenvironment of HCC. We discovered that immune cells with immunosuppressive effects in the immune microenvironment of HCC have a higher proportion of SMS-positive cells. Especially, liver promotes immune tolerance, preventing antigenic overload of certain substances absorbed from the gut (56). This study shows that a higher proportion of SMS-positive immune cells with immunosuppressive effects may promote the formation of stronger immunosuppressive environment in HCC, which is defined as “cold tumor” (57). Some SMS inhibitors could significantly enhance the therapeutic efficacy of polyamine depletion approaches, particularly in spermine-rich tumors (58–60). Tumor cells can consume a large amount of arginine in the tumor microenvironment, resulting in the lack of arginine in the tumor microenvironment, and the activation of anti-tumor immune cells will inevitably be inhibited (61). Therefore, inhibition of SMS to prevent arginine degradation in the tumor microenvironment is an attractive strategy to reactivate immune responses. Above findings indicate that inhibit the expression of SMS could result to the advancement of therapeutic strategies to weaken immunotherapy resistance in HCC and enhance checkpoint blockade therapy efficacy.

To get a full understanding about activity of SMS in HCC, we used TCGA-LIHC data to obtain DEGs by comparing the expression profiles of two groups (high and low expression of SMS). Interestingly, hepatitis virus infection, as a potential factor in the development of HCC, did not affect SMS expression. Furthermore, as indicated by GO and KEGG analysis, we discovered the SMS was correlated with chromosome segregation, mitotic nuclear division, organic and carboxylic acid biosynthetic process, etc.

Moreover, those important immune-related pathway, such as Intestinal immune network for IgA production, Fc gamma R-mediated phagocytosis, Antigen processing and presentation, Th1 and Th2 cell differentiation (**Figure 6E**), and cancer-related signaling pathways, such as MYC targets, G2 M checkpoint, activated E2F targets and PI3K-AKT-mTOR signaling (**Figure 6F** and **Extended Data Figure S3**), are closely related to the expression level of SMS in HCC when tested using GSEA, GSVA and ssGSEA. These results suggest that SMS overexpression is not only related to the immunosuppressive effect of tumor immune microenvironment, but also may be involved in the regulation of multiple tumor-related signaling pathways.

On the other hand, we analyzed genes significantly associated with SMS expression in HCC. We found some genes that were aberrantly expressed and associated with the overall survival of HCC, and these genes might constitute a network regulating the course of HCC with SMS. Pathway enrichment analysis of these genes showed that the regulatory network chiefly facilitated immune cell activity, which may further participate in the formation of immunosuppressive function in the immune microenvironment of HCC.

All data illustrates that SMS expression is widely drawn into the occurrence and development of cancer. In-depth study of the regulation mechanism of SMS expression and its regulatory network will contribute to clarify the pathogenic mechanism and the mechanism of immune escape in HCC.

Although this study expanded our knowledge of the connection between SMS and HCC, there were still some areas for improvement. Above all, the expression of SMS needs to be further verified in clinical samples. Next, the mechanistic details of the involvement of SMS in HCC development remain to be experimentally explored.

In conclusion, this study is the first to find that SMS expression is upregulated in HCC. We believe that SMS could be considered as a potential biomarker of poor prognosis and its expression level could provide a therapeutic reference for related HCC patients. Besides, the role of SMS in the tumor immune microenvironment makes it a potential target for immunotherapy in HCC.

## Data availability statement

The datasets presented in this study can be found in online repositories. The names of the repository/repositories and accession number(s) can be found in the article/**Supplementary Material**.

## Author contributions

LX and L-F-RQ conceived the project. LP, DW, LX, and L-F-RQ provided bioinformatics analysis and wrote the paper with input from all authors. All authors contributed to the article and approved the submitted version.

## Funding

This work was supported by Medical and Health Science and Technology Development Project of Shandong Province (2017WS822).

## Acknowledgments

We acknowledge Yuan Zhang and Min Wang for critical reading of our manuscript.

## Conflict of interest

Author LX is employed by Jiangsu Yifengrong Biotechnology Co., Ltd.

The remaining authors declare that the research was conducted in the absence of any commercial or financial relationships that could be construed as a potential conflict of interest.

## Publisher’s note

All claims expressed in this article are solely those of the authors and do not necessarily represent those of their affiliated organizations, or those of the publisher, the editors and the reviewers. Any product that may be evaluated in this article, or claim that may be made by its manufacturer, is not guaranteed or endorsed by the publisher.
