# Supplementary material for: Overexpression of SMS in the tumor microenvironment is associated with immunosuppression in hepatocellular carcinoma
Source: Front Immunol. 2022 Dec 5;13:974241. doi: 10.3389/fimmu.2022.974241 (PMC9760682; doi:10.3389/fimmu.2022.974241)
Supplement: Supplementary file 1 [file DataSheet_1.docx]

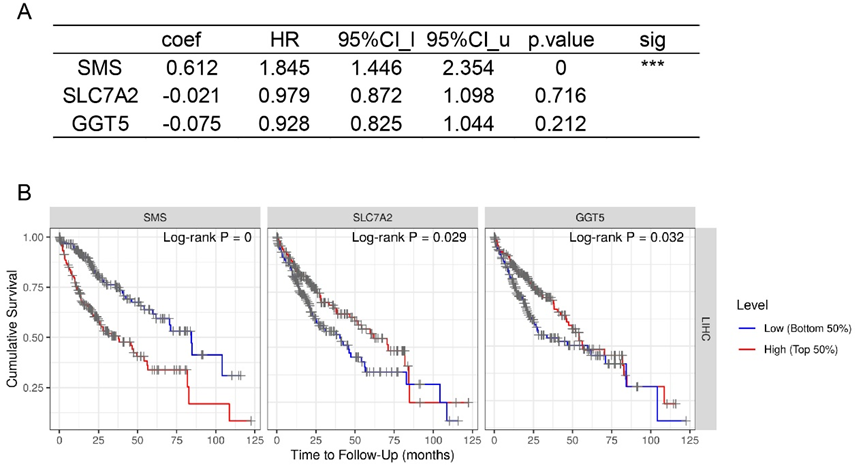


**Extended Data Fig. 1 (A)** The risk factors for overall survival of HCC were analyzed by multivariate COX regression analysis in TIMER 2.0 (***p < 0.001). (B) Kaplan-Meier plots of SMS, SLC7A2, GGT5 expression levels in HCC.


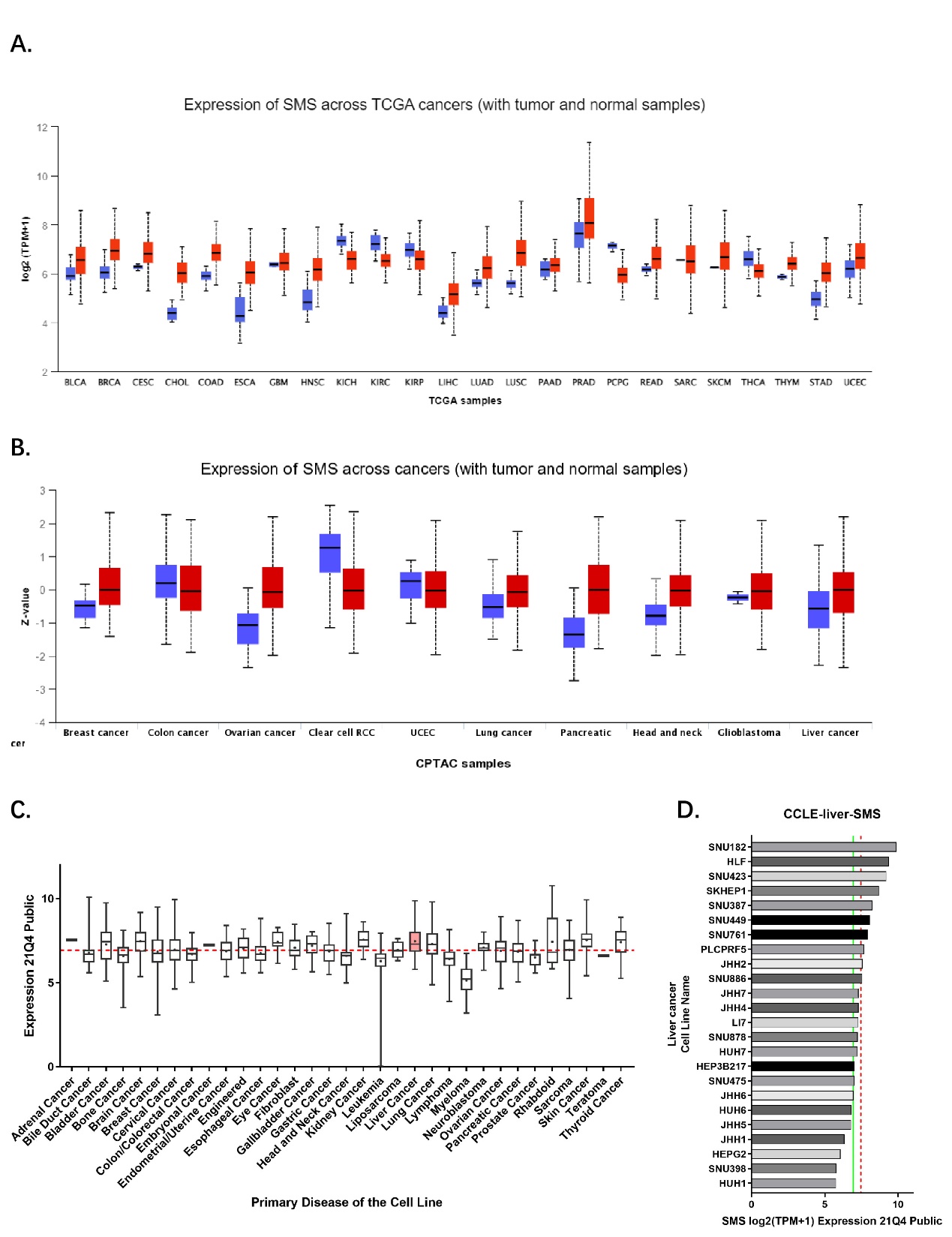


**Extended Data Fig. 2 (A-B)** The Expression pattern of SMS in Pan-cancer perspective. **(A)** The SMS mRNA expression was significantly overexpressed in almost all tumor types in TCGA database compared to the normal tissue, including bladder urothelial carcinoma (BLCA), breast infiltrating carcinoma (BRCA), cervical squamous cell carcinoma and adenocarcinoma (CESC), cholangiocarcinoma (CHOL), (COAD), (ESCA),pleomorphic glioma (GBM), Liver hepatocellular carcinoma (LIHC), lung adenocarcinoma (LUAD), (LUSC), Pancreatic cancer (PAAD), prostate cancer (PRAD), (READ), (SARC), skin melanoma (SKCM), thymic cancer (THYM), (STAD), endometrial cancer (UCEC) (p < 0.05). **(B)** SMS protein level was also significantly expressed in Breast cancer, Ovarian cancer, Lung cancer, Pancreatic, Head and neck, Glioblastoma, Liver cancer (p < 0.05). **(C-D)** Expression of the SMS in **(C)** pan-cancer and **(D)** Liver cancer according to CCLE.


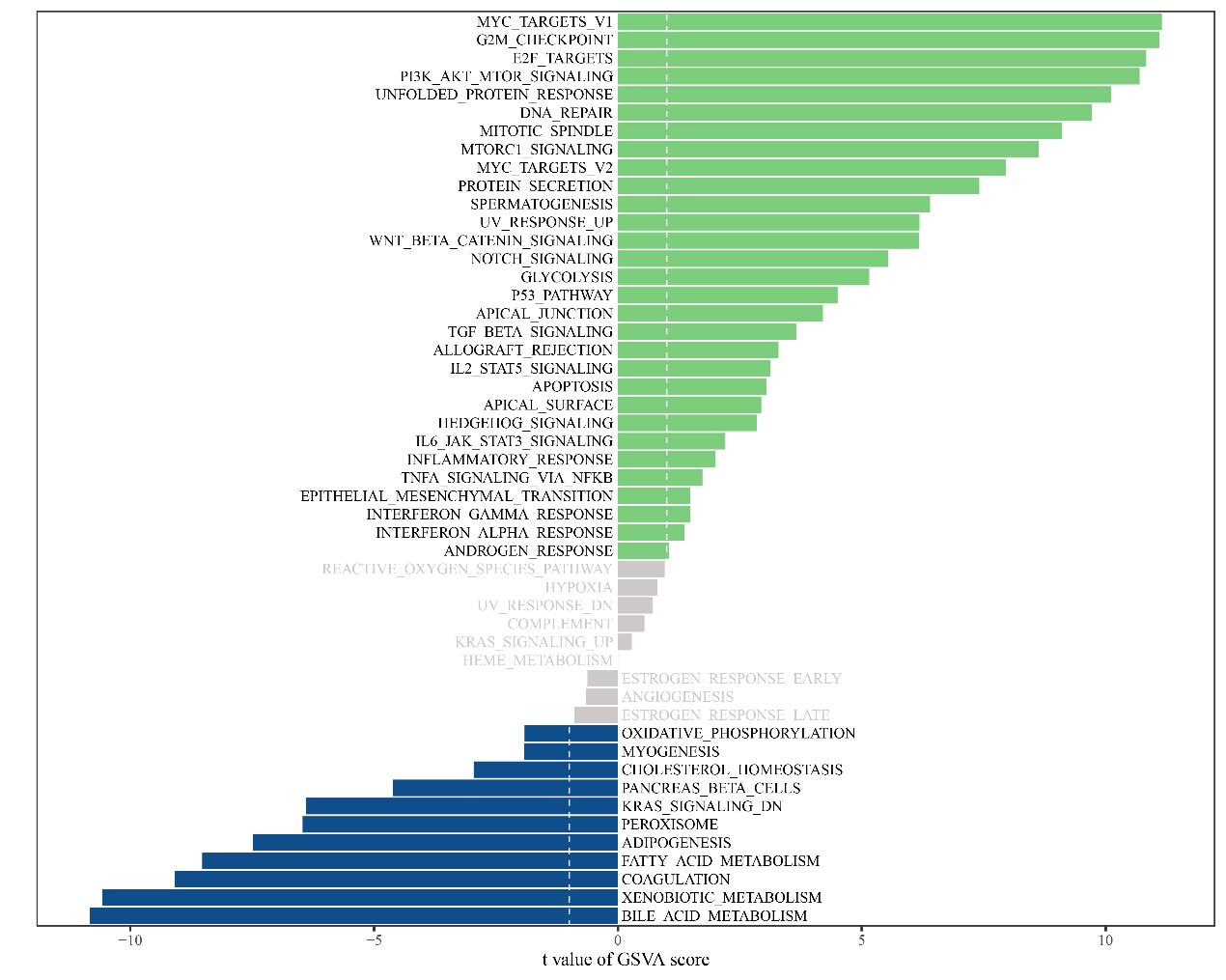


**Extended Data Fig. 3** GSVA of the high- and low-SMS expression clusters. The enrichment results of each pathway show the t-value, and the cut-off value is ±1, and the value between -1 and 1 is shown in gray.
